# Clinical assessment of upper limb impairments and functional capacity in Parkinson's disease: a systematic review

**DOI:** 10.1055/s-0043-1772769

**Published:** 2023-10-29

**Authors:** Tamine T. C. Capato, Rúbia Rodrigues, Rubens G. Cury, Manoel Jacobsen Teixeira, Egberto R. Barbosa

**Affiliations:** 1 Universidade de São Paulo, Faculdade de Medicina, Departamento de Neurologia, Centro de Distúrbios do Movimento, São Paulo SP, Brazil.; 2 Radboud University Medical Centre, Donders Institute for Brain, Cognition and Behavior, Department of Neurology, Nijmegen, The Netherlands.; 3 Universidade de São Paulo, Departamento de Neurocirurgia, São Paulo SP, Brazil.

**Keywords:** Parkinson Disease, Upper Extremity, Physical Therapy Modalities, Treatment Outcome, Rehabilitation, Freezing, Doença de Parkinson, Extremidade Superior, Modalidades de Fisioterapia, Resultado do Tratamento, Reabilitação, Congelamento

## Abstract

**Background** Parkinson's disease (PD) may progressively reduce the upper limb's functionality. Currently, there is no standardized upper limb functional capacity assessment in PD in the rehabilitation field.

**Objective** To identify specific outcome measurements to assess upper limbs in PD and access functional capacity.

**Methods** We systematically reviewed and analyzed the literature in English published from August/2012 to August/2022 according to PRISMA. The following keywords were used in our search: “upper limbs” OR “upper extremity” and “Parkinson's disease.” Two researchers searched independently, including studies accordingly to our inclusion and exclusion criteria. Registered at PROSPERO CRD42021254486.

**Results** We found 797 studies, and 50 were included in this review ( *n* = 2.239 participants in H&Y stage 1–4). The most common upper limbs outcome measures found in the studies were: (i) UPDRS-III and MDS-UPDRS to assess the severity and progression of PD motor symptoms (tremor, bradykinesia, and rigidity) (ii) Nine Hole Peg Test and Purdue Pegboard Test to assess manual dexterity; (iii) Spiral test and Funnel test to provoke and assess freezing of upper limbs; (iv) Technology assessment such as wearables sensors, apps, and other device were also found.

**Conclusion** We found evidence to support upper limb impairments assessments in PD. However, there is still a large shortage of specific tests to assess the functional capacity of the upper limbs. The upper limbs' functional capacity is insufficiently investigated during the clinical and rehabilitation examination due to a lack of specific outcome measures to assess functionality.

## Resumo

**Antecedentes** A doença de Parkinson (DP) reduz progressivamente a funcionalidade do membro superior. Não existe uma avaliação padronizada da capacidade funcional do membro superior na DP na área da reabilitação.

**Objetivo** Identificar medidas de resultados específicos para avaliar membros superiores na DP e avaliar capacidade funcional.

**Métodos** Revisamos e analisamos sistematicamente a literatura publicada de agosto/2012 a agosto/2022 de acordo com PRISMA. Usamos as seguintes palavras-chave “membros superiores” OU “extremidade superior” e “doença de Parkinson.” Dois pesquisadores fizeram a busca de forma independente, incluindo estudos de acordo com os critérios de inclusão e exclusão. Registro PROSPERO CRD42021254486.

**Resultados** Encontramos 797 estudos, 50 foram incluídos no estudo( *n* = 2.239 participantes no estágio 1–4 de H&Y). As medidas de resultados de membros superiores mais comuns encontradas foram: (i) UPDRS-III e MDS-UPDRS, para avaliar a gravidade e a progressão dos sintomas motores da DP (tremor, bradicinesia, e rigidez); (ii) Nine Hole Peg Test e Purdue Pegboard Test para avaliar a destreza manual; (iii) Teste da Espiral e Teste do Funil para provocar e avaliar o congelamento de membros superiores; (iv) Avaliação de tecnologia, como sensores vestíveis, aplicativos e outros dispositivos também foram encontrados.

**Conclusão** Encontramos evidências para dar suporte para as avaliações de deficiências de membros superiores na DP. No entanto, ainda há grande escassez de testes específicos para avaliar a capacidade funcional dos membros superiores. A capacidade funcional dos membros superior é insuficientemente investigada durante o exame clínico e de reabilitação devido à falta de medidas de resultados específicos para avaliar a funcionalidade.

## INTRODUCTION

Parkinson's disease (PD) is a neurodegenerative disease 1 2 diagnosed using clinical criteria, including bradykinesia, tremor, rigidity, and postural instability. 3 4 The clinical presentation can be multifaceted, including other motor and non-motor symptoms, differing among patients and subtypes. 5 The onset of symptoms is asymmetric, and since the early stages of PD, people experience a decrease in arm swing, 6 progressive speed reduction, and a decrease in the amplitude of the upper limb's repetitive movements. 7 Progressively, reduction of the upper limb's functional capacity generated by bradykinesia, tremor and rigidity may impact daily life activities, and freezing of upper limb (FOUL) episodes can be very disabling. 8

Various measurement instruments used to assess gait, freezing of gait (FOG), and balance in Parkinson's disease are reported. 9 10 However, only a few instruments are available for clinical assessing upper limb impairments in PD. 11 Usually, the test and scales do not provide sufficient information about the quality of task performance quality or the test target according to the intervention proposed. 12 13

Most of the tests and scales used in clinical practice and research measure the severity of PD motor symptoms including tremor, rigidity, bradykinesia, 14 manual dexterity, 15 16 and FOUL. 8 11 There is a gap of this instrument in clinical and rehabilitation fields that can reliably measure the upper limb functional capacity of a person with PD. Currently, there are no recommendations to assess upper limb functionality 12 13 17 18 19 and standardized upper limb impairments assessment in PD. 1 3 12 13 18 Here, in this systematic review, we aimed:

- to identify the available outcome measures to assess upper limb impairments in people with PD; and
- to identify specific outcome measures to assess functional capacity in PD.

## METHODS

### Registration

This study was registered at PROSPERO CRD42021254486.

### Search strategy and selection criteria

We reviewed systematically the literature published from August 2012 to August 2022 according to PRISMA 20 (checklist - supplementary material). We analyzed published studies from a systematic review in the PubMed, using the following search: ((“upper extremity”[MeSH Terms] OR (“upper”[All Fields] AND “extremity”[All Fields]) OR “upper extremity”[All Fields] OR (“upper”[All Fields] AND “limb”[All Fields]) OR “upper limb”[All Fields]) AND (“Parkinson disease”[MeSH Terms] OR (“Parkinson”[All Fields] AND “disease”[All Fields]) OR “Parkinson disease”[All Fields] OR “Parkinson s disease”[All Fields])) AND (“2012/08/22”[PDAT]: “2022/08/22”[PDAT]) AND.

### Eligibility criteria

#### *Inclusion criteria*

- We included all peer-reviewed studies that reported an upper limb or upper extremity assessment and rehabilitation interventions in PD; only studies published in English were included in this review;
- observational studies, experimental studies, and quantitative study designs, including clinical trials, meta-analyses, systematic reviews, and case reports published from August 2012 to August 2022 were also included in this review.

#### *Exclusion criteria*

- All studies that do not mention Parkinson's disease, and that do not present upper limbs assessments and rehabilitation interventions in methodology were excluded. Studies that used outcome measures and interventions that were not tested in PD patients were excluded.

### Study selection, study quality and risk of bias appraisal

Two researchers performed the search independently (RR and TC). A consensus meeting was held when needed to include or excluded accordingly our criteria. The researchers also investigated the trial's effect size, and an outcome measure cut-off that could be used as an effect size of the interventions in future trials or at least indicate motor disease severity or level of upper limb disability. The Preferred Reporting Items for Systematic Reviews and Meta-Analyses (PRISMA) 20 recommends the use of checklists to appraise study quality in systematic reviews. So, to evaluate the methodological quality of the included studies to determine whether the study was eligible for this review, and to reduce selection bias in the review, we used the Physiotherapy Evidence Database PEDro. 21 This database provides a good of information to evaluate the methodologic quality of the studies and risk of bias.

## RESULTS

**Figure 1:**
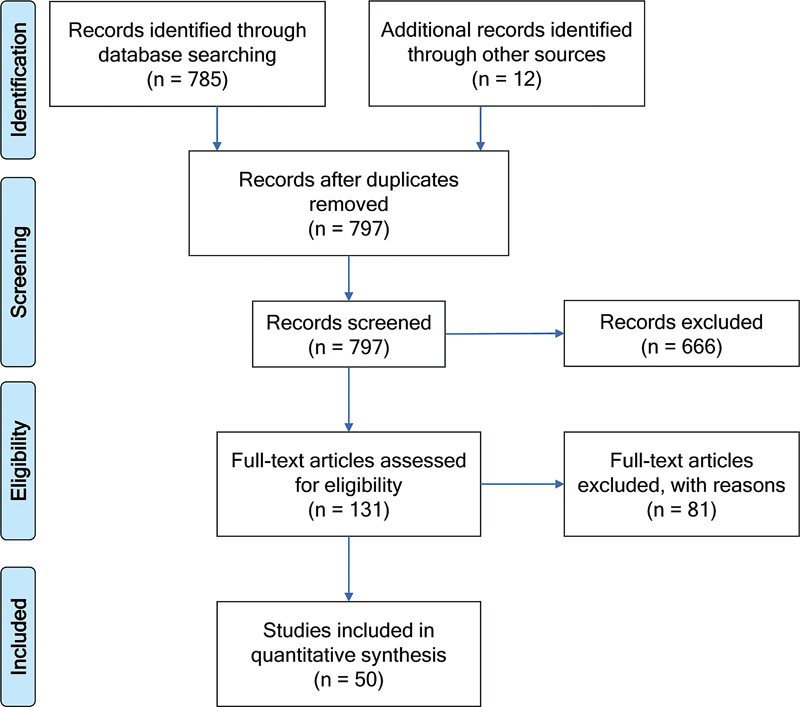
Flow diagram.

Initially, we found 797 studies, 785 in PubMed, and 12 in other sources ( Figure 1 ). According to the inclusion and exclusion criteria, 50 studies were included in this systematic review (2.239 participants in H&Y stage 1–4). Supplementary Material Table 1 ( https://www.arquivosdeneuropsiquiatria.org/wp-content/uploads/2023/10/ANP-230102-Supplementary-Material.pdf ) shows in detail the characteristics of studies assessing upper limb impairments in PD. In summary, we found many tests and scales which are used to assess upper limbs in PD:

- PD motor symptoms (severity and progression) can be assessed by Unified Parkinson's Disease Rating Scale 22 (UPDRS III) ( *n* = 28), 7 15 23 24 25 26 27 28 29 30 31 32 33 34 35 36 37 38 39 40 41 42 43 44 45 46 47 and MDS-Unified Parkinson's Disease Rating Scale 14 (MDS-UPDRS)( *n* = 18) 6 8 11 16 48 49 50 51 52 53 54 55 56 57 58 59 60 61 ; To access tremors we found Fahn-Tolosa-Marin Tremor Rating Scale 62 ( *n* = 5), 32 44 45 46 60 Tremor Rating Assessment Scale ( *n* = 1) 45 and Spiral test ( *n* = 2). 45 46 Other tests and scales were found to access bradykinesia such as the Finger tapping test ( *n* = 2) 38 63 and Patient Global Impression of Change ( *n* = 1), 46 Motor assessment scale ( *n* = 1), 61 Performance measure ( *n* = 1) 61 and Altering tapping performance ( *n* = 1). 36
- Manual dexterity can be assessed using the Nine Hole Peg Test ( *n* = 11) 15 16 23 31 47 48 49 51 61 64 65 and Purdue Pegboard Test ( *n* = 9) 16 24 33 44 50 61 63 64 66 ; Goal attainment scaling ( *n* = 2) 61 64 ; DextQ-24 ( *n* = 1) 48 ; Coin Rotation task ( *n* = 03) 16 48 49 ; Spiral (test *n* = 3) 8 38 45 ; Manual Ability Measure-36 questionnaire ( *n* = 1) 49 ; Functional Reach test ( *n* = 1) 27 ; Occupational Therapy Neurologic Assessment battery dexterity task ( *n* = 1) 50 ; Bimanual dexterity hardware and experimental setup ( *n* = 1) 52 ; Box & Blocks test ( *n* = 3) 16 30 47 ; Hand temporal and spatial parameters ( *n* = 1) 30 ; Edinburgh handedness Inventory ( *n* = 2) 11 53 ; Patient-Specific Index-Parkinson's Disease and Self-assessment Parkinson's Disease Disability Scale ( *n* = 1) 15 ; Functional motor task ( *n* = 1) 51 ; Disabilities of the Arm, Shoulder and Hand (DASH) ( *n* = 2) 16 29 ;
- FOUL have been assessed by using the Spiral test ( *n* = 3), 8 38 44 Funnel test ( *n* = 2). 8 53 We also found PD studies using technology to assess FOUL during alternating bimanual movements ( *n* = 2) 54 57 ; Finger tapping test ( *n* = 2) 44 55 ; handwriting and drawing patterns ( *n* = 1) 45 ;
- Technology has been used to assess the upper limb by using wearables sensors and apps or digital platforms. 44 67 Clinical-based, kinematic-based or kinematic have been assessed by using EMG ( *n* = 3). 6 68 69 Sensor units attached to the arms, hand and fingers can assess strength, movement power ( *n* = 3), 6 37 56 and arm swing ( *n* = 1). 40 O other outcomes were related to access Hand grip strength and finger measured by Dynamometer ( *n* = 05). 25 47 48 50 64 Power have been also assessed by using one repletion maximus (1RM)( *n* = 1) 26 ; and movement resistance in the wrist and finger muscles ( *n* = 1). 39

Only few studies included quality of life scales to verify the impact of upper limb impairments in daily life activities 35 46 48 61 70 ; We did not find specific outcome measures to assess the functional capacity of upper limbs in PD. We did not find in the studies a specific intervention effect size, or an outcome measure cut-off that can be used to indicate the level of upper limb disability.

## DISCUSSION

In this study, we aimed to identify the available outcome measures to assess upper limb impairments in people with PD; and specific outcome measures to assess functional capacity. Although we have found some evidence and useful outcome measures to assess of upper limb impairment assessments in PD, there is still a large shortage of specific tests to assess the functional capacity of the upper limbs in rehabilitation filed.

### Motor severity and disease progression

To assess the level of PD progression and motor severity of the disease, the UPDRS III and the MDS-UPDRS scales are widely used in many studies. 6 7 23 24 25 26 27 33 48 49 50 53 63 The MDS-UPSRS is the “gold standard” scale to access upper limbs tremors, rigidity and bradykinesia in clinical practice. By using specifically MDS-UPDRS domains (subscales), makes it possible to objectively evaluate upper limb resting tremor, and action tremor. It has also been reported that tremors can be assessed by using the Fahn-Tolosa-Marin Tremor Rating Scale 30 42 43 44 58 and Spiral test ( *n* = 2). 43 44 Our findings did not find any advantages in using the Fahn-Tolosa-Marin scale instead of MDS-UPDRS to access tremors. Future trials should investigate if one scale is superior to another to access tremors in the rehabilitation field. Other tests and scales can access bradykinesia, like the Finger tapping test. 36 61 Clinic-based experience and kinematic-based (EMG) assessments are also used to treat upper limb tremors in PD during BONT-A injection. 32 68

### Specific impairments which directly impact functional movements

The current guidelines for PD provide no strong recommendations to assess upper limb functionality, 12 13 17 18 19 and there is no consensus about outcome measures to assess specific impairments that directly impact functional movements. However, studies have shown that it is possible to clinical assess other upper limb impairments, such as manual dexterity. 16 It is well known that upper limb impairments can be very disabling during daily life activities. 16 55 On the other hand, the current measures can be potentially difficult to assess specific motor symptoms, such as rigidity can impact functional movements. 38 54 The scarcity of specific tests to evaluate upper limbs functional capacity leads to insufficiently investigation during a clinical examination and in research protocols.

The most common tests found in our search to assess manual dexterity were Nine Hole Peg Test 15 16 23 31 42 48 51 61 64 65 and Purdue Pegboard Test. 16 24 33 35 50 51 Both tests are effective and are the most assertive in evaluating the manual dexterity in PD. The Coin rotation test has been used in few studies on dexterity in PD. 29 48 The DextQ-24 is an interesting questionnaire to access dexterity in daily live activities and everyday tasks such as washing/grooming, dressing and others. 48 Both, Coin Rotation task and DextQ-24 and are easy and low cost to apply.

Our findings showed some outcome measures used for PD assessment in the studies searched were originally designed to access other diseases, such as “Test devaluation des Mem- bres Sup érieurs des Personnes Agées” (TEMPA). 71 Other examples are the Fugl-Meyer scale, 31 (it is a stroke-specific, performance-based impairment index) and the Jebsen Hand Function Test, originally developed to assess gross and hand function in patients with cervical spinal cord injury. 41

### Assess and provoke freezing of upper limbs (FOUL)

FOUL episodes can be very disabling during daily life activities. For this reason, FOUL should be objectively verified its presence, e.g., by evaluating the spiral-drawing task or the funnel task. 8 9 Interestingly, studies have shown that it is possible to provoke and access FOUL in a clinical setting. 11 55 72 It is important to highlight, the tests most used to assess FOUL: flexion and extension of the index finger, finger tapping (index finger on thumb collected by MDS-UPDRS) 23 55 and Funnel Task. 53 During these tasks, it is possible to identify that movements of very small amplitude, high frequency, and execution in dual tasks lead to more FOUL episodes. 11 53 57 Only one of these studies found a correlation between their intervention and the effects of transcranial direct current stimulation on FOUL during the funnel test. 53 Previous studies affirm the importance of a therapy based on specific FOUL goals to achieve significant improvements. 8 48 64 Moreover, lower limb motor control can be associated to upper limb control, being more easily incorporated into regular daily tasks. 73 The spiral and funnel test are an assessment tool for FOUL during a task, which can be an important marker of the development of the pathology during the test time, as well as providing feedback for both clinicians and patient. 38 74 These studies align with the findings of, 49 emphasizing the need to consider the specificity of the proposed task, optimizing gains by including dual-task tasks exercises. 6 34 56 73 Therefore, FOUL deserves tailored treatment, and patients must be educated about compensation strategies by a physiotherapist with expertise in PD management. 8

It is important to emphasizes the importance of dual-task exercises 23 and the use of rhythmic cues to ameliorate FOUL. 8 9 Prior studies warned about the possibility of patients affected by FOUL and FOG, becoming dependent on rhythmic cues and highlighting how technology can be allied in assessing the delivering cues, optimizing their effectiveness. 47 It is still unclear whether the FOUL and FOG share the same physiological mechanism and the scarcity of specific tests to evaluate FOUL may limit the therapeutic approach. 61

In this context, technology, through applications, can be an important tool for evaluating the commitment of the upper limbs, as well as an ally in the therapeutic approach and, a promising strategy in the area. 6 32

### Technology in upper limb assessment

Our finds showed the use of technology to measure arm swing, and arm swing magnitude. Perhaps it can be useful for differentiating PD patients in early stage from healthy individuals. 58 In addition, some devices are portable and have been used to assess the impairments of the upper limbs in the clinical setting, and perhaps at home. Bradykinesia have been assessed on telemedicine with touch-pad to evaluation of alternating tapping performance using a touch-pad handheld, however the implementation of this method requires more research. 36 An appropriate assessment will prevent false-negative results and allow the phenomenon to be identified and treated. So far, there is no strong evidence that these methods have any advantage over assessment traditional methods. Therefore, despite they are promising, future studies should evaluate the ability of these technologies to complement traditional upper limb clinical exams to optimize pharmacological, surgical, and non-pharmacological interventions.

### Limitations

Limitations of this review included possible bias due to the lack of measures currently in the development or studies that include motor fluctuations such as dyskinesias and individual participation in daily life activities. In our inclusion, there was heterogeneity across many studies regarding the outcome measures used to assess the same variable, which made it challenging to compare all the studies' results. In addition, the small samples and variability of methods were often difficult to assess. Therefore, it was not possible to perform a meta-analysis, a specific intervention effect size, and indicate an outcome measure cut-off that can be used to indicate the level of upper limb disability. Finally, we cannot make any statement about the technology to assess functional capacity and its use in the late stages of PD. Using technology during clinical measurements to monitor upper limb impairments in a clinical setting and hoe-based can be a promising strategy. However, large clinical trials should confirm these findings.

### Clinical implications

In our opinion, the upper limbs' functional capacity is under investigation during the clinical and rehabilitation examination due to a lack of specific outcome measures in the movement disorders field. Therefore, a proper outcome measure may be important not only as a marker of the progression of the pathology but also as a basis for therapeutic interventions that improve the quality of life of individuals. 71 For various reasons, we strongly believe that standard evaluation of upper limb functional capacity can be an essential element in PD clinical management. First, we can use this kind of assessment to develop and follow a pharmacological, non-pharmacological, and surgical treatment program in all disease stages of PD, to be consistently referred and to select the interventions according to the upper limb deficits. Second, to quantify motor deficits before and to demonstrate the results after clinical and surgical interventions including neuromodulation and rehabilitation program to optimize these treatments since the early stages of PD up to advanced stages. Finally, a functional capacity assessment could detect, monitor and support the decision of whether a person with PD can continue independently, work, and determine when tasks work-related should be adapted or discontinued. More studies are needed to verify this concept.

In conclusion, we found evidence to support upper limb impairment assessments in PD. However, there is still a large shortage of specific tests to assess the functional capacity of the upper limbs in movement disorders rehabilitation field. Further studies should investigate technological advances to refine and support outcomes of assessing upper limb impairments.
