# Supplementary material for: Clinical assessment of upper limb impairments and functional capacity in Parkinson's disease: a systematic review
Source: Arq Neuropsiquiatr. 2023 Oct 29;81(11):1008–15. doi: 10.1055/s-0043-1772769 (PMC10689111; doi:10.1055/s-0043-1772769)
Supplement: Supplementary file 1 — Supplementary Material [file 10-1055-s-0043-1772769-s230102.pdf]

Supplementary Material Table 1 Overview, characteristics and outcomes of studies which to assess upper limbs impairments in Parkinson's disease

Author	Objective	UL impairments as an Inclusion Criteria	Upper limb impairments assessed	Age (Mean)	H&Y	Disease duration (years) mean	Sample	Medication state (ON/OFF)	Intervention	Follow-up	Outcomes to access Upper limb	Study design	PEDro (0/10)
Braun Janzen, et al. 2019[23]	To examined whether rhythmic finger movements would prime the motor system and modulate gait.	NO	Tremor; Rigidity; Bradykinesia; Dyskinesias; Dystonia	Finger tapping: 68.4; Arm swing: 64.2; CG: 67.2	1–2	Finger tapping: 4.8; Arm swing: 7.7; CG: 11.7	PD Finger tapping: 11; PD Arm swing: 14; PDCG: 12.	ON	Rhythmic Auditory Stimulation training of arm or finger movements would modulate gait speed.	No follow-up	MDS-UPDRS (III and IV); NHPT	Randomized Controlled Trial	Clinical Trial (9/10)
Cabrera-Martos et al. 2019[64]	To evaluate the effects of an intervention based on a specific set of goals on goal attainment, manual dexterity, hand grip strength and finger prehension force compared with a standardized approach in PD.	YES	Manual Dexterity, Hand grip strength and finger prehension force.	PDEG: 69.4<br>PDCG: 71.7	2–3	PDEG: 6.4<br>PDCG: 7.1	PDEG: 25<br>PDCG: 25	ON	PDEC: intervention focused on task components that involved goals proposed by participants<br>PDCG: standard intervention focused on impairments in range of motion, grasp and manipulation	4 weeks	Goal attainment scaling; NHPT; PPT; Hand grip strength and finger prehension force JAMA Dynamometer;	Parallel single-blinded Randomized Controlled Trial	Clinical Trial (8/10)
Momin et al. 2018[24]	To investigate the acute effects of various STN-DBS frequencies (40–160 Hz, 40 Hz intervals) on UL motor function.	Yes	Tremor; Bradykinesia	60.4	Not mentioned	11.8	PD= 20	OFF	STN-DBS	No follow-up	UPDRS-III; UL-UPDRS-III; PPT	Double-blind Randomized Controlled trial	No records found
Jitkritsadaku et al. 2017[7]	To investigate the efficacy of EMS, delivered using Tremor's glove, as a treatment of resting hand tremor.	YES	Tremor	PDEG: 63.4<br>Sham G:64.3	2–3	PDEG: 7.9<br>Sham G: 6.6	PDEG:15<br>PDCG:15	ON and OFF	Tremor's glove-an innovative electrical muscle stimulation (EMS)	1 month	UPDRS III (tremor scores)	Double-blind Randomized Controlled trial	Clinical Trial (5/10)
Vanbellingen et al. 2017[48]	To investigate the effectiveness of a home-based dexterity program on fine motor skills in PD.	NO	Manual Dexterity; Strength, PD motor symptoms; Tremor; Rigidity; Bradykinesia; ADL; Dexterity-related; isometric hand strength	PDEC: 67.15<br>PDCG: 68.1	1–4	PDEC: 6.1<br>PDCG: 6.35	PDEC: 52<br>PDCG: 51	ON	Home-based dexterity program (HOMEDEXT) or Thera-band program	12 weeks	MDS-UPDRS II and III; NHPT; PDQ-39; DextQ-24; CRT; Isometric hand strength was measured using the JAMA Dynamometer;	Observer-blinded Randomized controlled trial	Clinical Trial (8/10)
Allen et al. 2017[49]	To investigate if exergames targeting the UL improve arm and hand activities and impairments and to establish the acceptability and feasibility of these games in PD.	NO	Manual Dexterity; Manual Ability	PDEG: 67.5<br>PDCG: 68.4	Not mentioned	PDEG: 7.9<br>PDCG: 8.7	PDEG: 19<br>PDCG: 19	ON	PDEG: Home-based UL exergame intervention<br>PDCG: Maintain usual activities for 12 weeks	12 weeks	MDS-UPDRS; MDS-UPDRS (UL scores); NHPT; CRT; activities and impairments, including the tapping test [speed (taps/60s), and error (weighted error score/speed)].Manual Ability Measure-36 questionnaire	Randomized controlled trial	Clinical Trial (8/10)
David et al. 2016[25]	To examined the spatiotemporal changes in agonist and antagonist muscle activity following 24 months of progressive resistance exercise and the combined relationship between spatiotemporal muscle activity and strength measures and upper limb bradykinesia.	NO	Bradykinesia; Strength	Progressive Resistance Exercise Training (PRET): 59<br>Modified Fitness Counts (mFC): 58.6	1–3	Progressive Resistance Exercise Training (PRET): 6.5<br>Modified Fitness Counts (mFC): 6.5	Progressive Resistance Exercise Training (PRET): 24.<br>Modified Fitness Counts (mFC): 24	ON and OFF	Progressive resistance exercise training (PRET) with a Nonprogressive exercise intervention, modified Fitness Counts (mFC)	24 months	UPDRS-III; Movement velocity and isometric strength was a single-degree-of-freedom manipulandum that consisted of a metal bar with a handle (combined moment of inertia 0.14 kg·m2) freely rotating in a horizontal plane around a pivot centered at the elbow joint.	Parallel<br>Blinded<br>Randomized clinical trial	Clinical Trial (5/10)
Ni, Signorile et al. 2016[27]	To compare the effects of power training (PWT) and a high-speed yoga program on physical performances in PD; to test the hypothesis that both training interventions would attenuate PD symptoms and improve physical performance.	NO	PD motor symptoms; Bradykinesia	PD	1–3	PWT: 6.6<br>Yoga: 6.9<br>CG: 5.9	PD PWT: 14<br>PD Yoga: 12<br>PD CG:10	ON	Two high-speed exercise interventions (specifically designed yoga program and PWT) were given for 12 weeks (twice a week), and 1 no exercise control group.	No follow-up	UPDRS III; Functional Reach Test	Randomized Controlled Trial	Clinical Trial (6/10)
Ni, Mooney et al. 2016[26]	To examine the effects of a high-speed power yoga program on bradykinesia, muscle function and quality of life in PD; to examine the relationship of clinical measures of bradykinesia to muscle power.	NO	Bradykinesia; Rigidity; Strength; Power	PD Yoga: 71.2<br>PDCG: 74.9	1–3	PD Yoga: 6.9<br>PDCG: 5.9	PD Yoga: 14<br>PDCG: 12	ON	Power yoga program (YOGA), and no exercise control group.	3 months	UPDRS III; UPDRS UL scores; One repetition maximums (1RM) and peak powers on biceps curl,	Randomized controlled trial<br>Pilot study	Clinical Trial (5/10)
Mateos-Toset al. 2016[50]	To evaluate the effects on manual dexterity, hand grip, and pinch strength of a single intervention focused on hand exercises in PD.	NO	Manual dexterity; Hand grip ; Pinch Strength	PD: 72.6<br>CG: 69.9	2–3	PD: 6.6<br>CG: 7.1	PD: 30<br>CG: 30	ON	EG: an exercise session focused on hand training; CG: active upper limb range of movement exercises	No follow-up	MDS-UPDRS; PPT; Chessington Occupational Therapy Neurologic Assessment Battery dexterity task (COTNAB); JAMA Dynamometer (Hand grip and pinch strength)	Blinded Randomizedcontrolled trial	Clinical Trial (7/10)

(Continued)

 © 2023. Academia Brasileira de Neurologia. All rights reserved.

Supplementary Material Table 1 (*Continued*)

Author	Objective	UL impairments as an Inclusion Criteria	Upper limb impairments assessed	Age (Mean)	H&Y	Disease duration (years) mean	Sample	Medication state (ON/OFF)	Intervention	Follow-up	Outcomes to access Upper limb	Study design	PEDro (0/10)
Pazzaglia et al. 2020[29]	To compare a 6-week virtual reality (VR) rehabilitation program with a conventional rehabilitation program in PD	NO	Disabilities of the Arm, Shoulder and Hand; PD motor symptoms	PDEG: 72<br>PDCG:70	Not Mentioned	PDEG: 7.4<br>PDCG 4	PDEG: 26<br>PDCG: 25	ON	PDEG: 6-week virtual reality (VR) rehabilitation program; PDCG: conventional rehabilitation program	6 weeks	UPDRS III; Disabilities of the Arm, Shoulder and Hand (DASH)	Single-blinded, randomized, controlled trial.	Clinical Trial (5/10)
Paul et al. 2020[51]	to determine whether dopamine replacement medication (i.e., levodopa) affects: learning of a novel UL task, decrements in skill following withdrawal of practice, the rate of learning, and the transfer of movement skill to untrained UL tasks compared with training "off" medication, in PD		Manual dexterity;	PDON LEVOPODA: 71.7<br>PDOFF LEVODOPA:70.31	2	PDON LEVOPODA: 5.3<br>PDOFF LEVODOPA: 3.4	PDON LEVOPODA: 12<br>PDOFF LEVODOPA: 11	ON and OFF	To practiced 10 blocks of five trials of a functional motor task with their non-dominant UL over three consecutive days (acquisition period), followed by a single block of five trials two and nine days later. Participants were also assessed "on" levodopa with two transfer tasks (the nine-hole peg test and a functional dexterity task) prior to any practice and nine days after the end of the acquisition period.	followed by a single block of five trials two and nine days later	MDS-UPDRS; NHPT; Functional motor task;	Randomized clinical trial	No records found
Jansen et al. 2021[52]	To determine the effects of high intensity lower extremity exercise on motor control patterns underlying a manual dexterity task.	Manual Dexterity; Grip and load forces	NO	PDFE: 63.5<br>PDVE: 62	2	PDFE: 3.7<br>PDVE: 3.3	PDFE: 14<br>PDVE:15	OFF	PD participants completed eight weeks (24 sessions) of high intensity aerobic exercise under forced or voluntary exercise (FE or VE) modalities. Grasping forces for each limb were quantified during a functional bimanual dexterity task.	8 weeks	MDS-UPDRS III; Bimanual dexterity hardware and experimental setup	Randomized Clinical Trial	No records found
Cikajlo et al. 2019[30]	to investigate the functional improvements, motivation aspects and clinical effectiveness when using immersive 3D virtual reality versus non-immersive 2D exergaming.	YES	Hand temporal and spatial parameters	PDEG: 71.3<br>PDCG: 67.6	2–3	7.1	PDEG: 10<br>PDCG: 10	ON	PDEG: using immersive 3D virtual reality<br>PDCG: non-immersive 2D exergaming	3 weeks	UPDRS; Box & Blocks test (BBT);<br>Hand temporal and spatial parameters Total time from the first touch with the virtual cube to the last cube inserted in the virtual chest	A parallel study.	Clinical Trial (4/10)
Picelli et al. 2014[31]	To evaluate whether robot-assisted arm training might improve upper limb function in PD.	NO	Dexterity;<br>PD motor symptoms;<br>UL motor ability to perform selective movements	Not Mentioned	2,5–3	7.1	PD: 10	ON	Robotic arm training (RAT)	2 weeks	UPDRS - I; II; III; NHPT; Fugl-Meyer scale	Pilot study	No records found
Broeder et al. 2019[53]	To investigate the effects of tDCS on FOUL and writing performance when applied while participants perform a freezing provoking task.	YES	PD motor symptoms;<br>Handedness;<br>FOUL	PD: 63.2<br>HC:64.5	2	PD: 6.9	PD: 10<br>HC: 10	ON	Transcranial (tDCS) over the primary motor cortex (M1); pplied with motor performance lasted 20 min. On-line motor performance consisted of three bouts (i.e., two runs cued and one run without visual cues) of writing several sequences of letters (lasting each ~3 min) followed by execution of the funnel task	No follow-up	MDS-UPDRS III; Edinburgh handedness Inventory; Funnel task.	Pilot study	No records found
Samotus et al. 2022[68]	To review our clinical experience for treating upper-limb Essential tremor (ET) and PD tremor using BoNT-A.	YES	Tremor	PD; 72.9<br>ET:72.9	Not Mentioned	Not mentioned	PD: 45<br>ET: 68	ON	An expert injector versus kinematic-based BoNT-A injection	7 years	Clinic-based, kinematic-based, or kinematic- (EMG)	Longitudinal restrospective study	No records found
Samotus et al. 2017[8]	To demonstrate that BoNT-A therapy coupled with kinematic guidance can provide efficacious outcomes for upper limb tremor with minimized unwanted weakness.	YES	Tremor	Not mentioned	1–3	Not mentioned	PD: 28<br>ET: 24	ON	Six serial BoNT-A treatments every 16 weeks	96 weeks	UPDRS; FTM; QUEST	Open label no randomized Trial phase II pilot study protocol	No records found
Van den noort et al. 2017[6]	To explore and demonstrate the applicability of quantifying hand motor symptoms in PD with the PowerGlove and an	YES	Tremor;<br>Bradykinesia;<br>Rigidity	not mentioned	Not mentioned		PD: 4	ON and OFF	PowerGlove System calibration	No follow-up	MDS-UPDRS; Upper limb Sensor units were attached to the hand and fingers; to be able to measure kinematics.	A Proof-of-Principle Study	No records found

 © 2023. Academia Brasileira de Neurologia. All rights reserved.

Supplementary Material Table 1 (*Continued*)

Author	Objective	UL impairments as an Inclusion Criteria	Upper limb impairments assessed	Age (Mean)	H&Y	Disease duration (years) mean	Sample	Medication state (ON/OFF)	Intervention	Follow-up	Outcomes to access Upper limb	Study design	PEDro (0/10)
	additional force sensor in a clinical setting.												
Eggers, et al. 2015[33]	To investigate whether a period of continuous theta burst stimulation (cTBS) over the supplementary motor area (SMA) induces cortical plasticity and thus improves bradykinesia in PD in ON and OFF.	NO	Bradykinesia<br>Manual Dexterity;	PDON: 65<br>PDOFF: 61	1–3	PDON: 6<br>PDOFF: 17	PDON: 13<br>PDOFF: 13	ON and OFF	Continuous TBS over the SMA and sham	No follow-up	UPDRS III; PPT	Parallel Exploratory Randomized controlled blind trail	No records found
Park et al. 2021[65]	To evaluate the effects of a drum playing intervention with rhythmic cueing on UL motor control and attention control in PD.	NO	Manual Dexterity	PDEG: 61.6<br>PDCG: 63.1	2–3	PDEG: 5.6<br>PDCG: 4.8	PDEG: 8<br>PDCG: 8	ON	The intervention consisted of the bimanual exercise of drum playing to strengthen motor control with and without cue.	12 weeks	NHPT; Two task conditions: drum tapping with and without cueing.	Randomized Clinical trial	No records found
Proud et al. 2013[61]	To investigate the frequency of physiotherapy and occupational therapy assessment of the upper limb (UL) in PD; to identify the impairments and activity limitations assessed and the methods used	NO	Tremor, Rigidity, Bradykinesia, and Dyskinesia; Strength; Sensibility	Not Mentioned	Not Mentioned		PT: 122<br>OT: 68	ON	Using a self-report questionnaire, we surveyed physiotherapists and occupational therapists to investigate their assessment practices.	No follow-up	UPDRS; PPT; NHPT; Motor Assessment Scale; Canadian Occupational Performance Measure; Goal Attainment Scale;<br>PDQ-39; Jebsen Test of Hand Function.	Qualitative study	No records found
Nieuwboer et al. 2009[54]	To identify whether freezing episodes occur during a bimanual rhythmic task and whether this is related to FOG severity; to determine whether spatiotemporal and coordination measures differed in controls, freezers and non-freezers in different amplitude and speed conditions.	NO	Bimanual rhythmic task speed/<br>Bradykinesia.<br>FOUL; Dyskinesia	NA	NA	NA	PT:122<br>OT:69	NA	Bimanual rhythmic task on a height-adjustable chair in front of two digitizing tablets with a pen-like stylus in the right and left hand (LC20-TDS Terminal Display Systems); to perform alternating movements in a rhythmical fashion at small (2cm) and large (4cm) amplitudes and at self-determined normal and maximal speed.	No follow-up	MDS-UPDRS; Bimanual task digitizing tablets (alternating movements in a rhythmical fashion at small (2cm) and large (4cm) amplitudes and at self-determined normal and maximal speed.<br>Video taped	Observational study	No records found
Heremans et al. 2015[11]	To investigate whether FOUL and other kinematic abnormalities during writing are provoked by (i) gradual changes in amplitude or by (ii) sustained amplitude generation in patients with and without freezing of gait.	Yes	Writing speed/<br>Bradykinesia;<br>FOUL	PD +FOG: 64.5<br>PD -FOG: 64.6<br>HC: 65.8	2	PD +FOG: 10.3<br>PD -FOG: 5.8<br>HC: NA	PD +FOG: 17<br>PD -FOG: 17<br>HC: 10	ON and OFF	Writing data were recorded on a touch-sensitive writing tablet	No follow up	MDS-UPDRS III; Edinburgh handedness inventory.	Observational study	No records found
Messa et al. 2019[34]	To investigate the effect of an upper-limb exercise, conceptually comparable with the forced exercise (FE), in PD.	.No	Pain;<br>PD motor symptoms	PD FE: 69.1<br>CG: Not Mentioned	3	PD FE:5<br>CG: NA	PD FE: 10<br>CG: 16	ON	Upper limb forced exercise	8 weeks	UPDRS-III	Observational study	No records found
Nowinski et al. 2016[35]	To present the results of Neuro-QoL validation in PD.	NO	UL Function–Fine Motor (fine motor and ADL), PD motor symptons; Change in function	65.5	1–4	7.1	PD: 120	ON	Assessments included Neuro-QoL and general and PD-specific validity measures.	6 months	UPDRS; Neuro-QoL; PDQ-39; Change in function/HRQoL	Prospective study	No records found
Samotus et al. 2018[70]	To demonstrate thatEssential Tremor (ET) and PD participants who already received beneficial unilateral bont-a therapy can transition to bilateral BoNT-A injections for their upper limb tremor for further improvement.	YES	Tremor	Not mentioned	Not mentioned	Not mentioned	ET: 5<br>PD: 2	On and off	BoNT-A injections	18 weeks	FTM; QUEST; **QoL**-Quality of life;	Open-label pilot trial	No records found
Memedi et al. 2013[36]	To development and evaluation of a method for enabling quantitative and automatic scoring of alternating tapping performance (ATP) using a touch-pad handheld computer	NO	Bradykinesia	PD: 65<br>HC: 6110	Not Mentioned		PD: 95<br>HC: 10	ON	Touchpad to evaluation of alternating tapping performance (ATP) using a touch-pad handheld computer designed for telemedicine	36 months	UPDRS; Alternating Tapping Performance	Open longitudinal Study	No records found

(*Continued*)

 © 2023. Academia Brasileira de Neurologia. All rights reserved.

Supplementary Material Table 1 (*Continued*)

Author	Objective	UL impairments as an Inclusion Criteria	Upper limb impairments assessed	Age (Mean)	H&Y	Disease duration (years) mean	Sample	Medication state (ON/OFF)	Intervention	Follow-up	Outcomes to access Upper limb	Study design	PEDro (0/10)
	designed for telemedicine in PD.												
Deval et al. 2017[55]	To determine the occurrence of episodic FOUL and FOG during MDS-UPDRS in the presence and absence of a metronome set to 4 Hz; to test the reliability of these phenomena between 2 sessions and their relationship with the severity of FoG in advanced PD.	NO	FOUL	PD +FOG: 69	2–3	PD +FOG:13	PD + FOG:15	ON	Finger tap test, (i) as big and as fast as possible (ii) as big as possible in time with an auditory cue (a metronome set to 4 Hz). The order of the two conditions was pseudo-random. At least 30 productions on each body side were recorded.	No follow-up	MDS-UPDRS (items 3.4 and 3.8); FOUL during Finger Tap test with and without auditory cues;<br>Kinematic parameters were recorded using a VICON 3D motion analysis system	Observational study	No records found
Jo et al. 2016[37]	To investigate the unintentional drift in total force and in sharing of the force between fingers in two-finger accurate force production tasks performed without visual feedback in PD and healthy controls; to test whether that adaptation to the documented loss of action stability could lead to faster force drop in PD.	YES	Strength;	PD: 63.1<br>HC: 63.3	2	PD: 2.2<br>HC: NA	PD: 10<br>HC: 10	ON	PD patients and healthy controls performed accurate constant force production tasks without visual feedback by different finger pairs, starting with different force levels and different sharing patterns of force between the two fingers.	No follow-up	UPDRS- III; Tasks with two fingers pressing on individual force sensors	Exploratory study	No records found
Pradhan et al. 2015[56]	To use a novel force sensor system to characterize grip force modulation, including force, temporal, and movement quality parameters, during a fine motor control task in early stage of PD.	NO.	Strength; Precision and Power grasp	PD: 63.7<br>CG: 63.8	1–2	NA	PD: 14<br>CG: 14	OFF	To twist each cap in clockwise and counterclockwise directions; 20 trials in each direction for the precision grasp and for the power grasp under single-task conditions, where only the fine motor control task was performed. A fine motor control task that was continuous and required rapid switching of movement direction.<br>This protocol was repeated under dual-task conditions, during which participants were asked to perform a concurrent cognitive task.	No follow up	MDS-UPDRS; Time to peak force; Movement time; movement arrest	Case-control study	No records found
Williams et al. 2013[57]	To investigate how manipulations of movement amplitude and cadence affect UL coordination as measured by the phase coordination index (PCI) and the number of FOUL events.	NO	FOUL	PD +FOG: 70.8<br>PD -FOG: 67.6<br>HC: 68.4	1–3	Not mentioned	PD +FOG: 16<br>PD -FOG: 12<br>HC: 19	OFF	15-second trials of alternating bimanual movements between targets under four conditions: Baseline; Fast; Small; SmallFast. Kinematic data were recorded and analyzed for PCI and FO-UE events.	No follow up	MDS-UPDRS; 15-second trial of Motion Analysis Corporation	Observational Clinical Study	No records found
Ospina et al. 2018[58]	To assess the differences in arm swing between PD patients and healthy participants and to investigate the possible effects of aging on these differences	NO	Arm movement; Arm swing; PD motor symptoms;	PD: 67<br>CG: 67	1–2	Not mentioned	PD: 25<br>CG: 25	ON	To evaluated gait-linked arm swing changes in early-stage PD patients using an RGB-D camera coupled to a signal processing software	No follow-up	MDS-UPDRS	Observational, single-center study	No records found
Saunders-Pullman et al. 2008[38]	To assess the validity of spiral analysis in measuring PD severity in the early stages of disease, by evaluating its cross-sectional association with overall motor UPDRS, as well as subscores for bradykinesia, rigidity, and tremor.	NO	Tremor; Rigidity; Bradykinesia;	69.9	Not Mentioned	61.5	PD: 74	ON	Handwritten spirals were acquired using a digitizing tablet (Intuos 2, Wacom Technology, Vancouver, WA) connected to a microcomputer using proprietary software written	No follow-up	UPDRS III; Finger tap test; hand movements; rapid alternating movements; Spiral test	Longitudinal observational blinded study	No records found
Cavallo et al, 2019[59]	To analyze a comprehensive experimental protocol for a complete motor evaluation of upper limbs in PD using six exercises taken from MDS-UPDRS III and, therefore, increasing the number	YES	PD motor symptoms; Tremor; pronation/ supination; thumb-forefinger tapping	PD: 65.2<br>Idiopatic Hyposmia: 66<br>HC: 67.9	1–2	Not mentioned	PD: 30<br>Idiopatic Hyposmia: 30<br>HC: 30	ON	To use a wearable inertial device, named SensHand V1 to acquire motion data from the upper limbs during the performance of six tasks selected by MDS-UPDRS III.	No follow-up	MDS-UPDRS III	Obsrvational study Experimental protocol	No records found

 © 2023. Academia Brasileira de Neurologia. All rights reserved.

Supplementary Material Table 1 (*Continued*)

Author	Objective	UL impairments as an Inclusion Criteria	Upper limb impairments assessed	Age (Mean)	H&Y	Disease duration (years) mean	Sample	Medication state (ON/OFF)	Intervention	Follow-up	Outcomes to access Upper limb	Study design	PEDro (0/10)
	of exercises with respect to previous works; To evaluate a wide set of features extracted from the kinematic analysis that enable evaluation of spatial, temporal, and frequency parameters, To investigate the most suitable ML approach for motor assessment of upper limb performance in PD by using three supervised classifiers (i.e., SVM, RF, and NB).												
Proud et al. 2021[15]	To describe NHPT performance for the more affected hand and examined the construct validity of these pegboard scores in mild to severe PD	NO	Manual Dexterity;	68.4	1–4	5.2	PD: 582	ON	Baseline trial data were used for this analysis. Participants were assessed in their homes and were asked to nominate their more affected hand for the pegboard testing.	No follow-up	UPRDS; NHPT; Patient-Specific Index-Parkinson's Disease and Self-assessment Parkinson's Disease Disability Scale	Observational study analyzed baseline data from a cluster-randomized controlled trial	No records found
Chan et al. 2018[60]	To present a new method to quantify tremor in angular parameter whereby using kinematic sensors, the multi–degrees–of–freedom (multi–dof) coupled relative displacement of body segments can be computed.	YES	Tremor	Not Mentioned	Not Mentioned	Not Mentioned	PD: 38	ON	In–laboratory test was performed to determine the validity and reliability of the developed tremor measurement system. Three–dimensional (3D) orientation of each hand–arm segment was measured using three units of microelectronic mechanical system (MEMS) attitude and heading reference system.	No follow-up	MDS-UPDRS; FTM; Washington Heights–Inwood Genetic Study of Essential Tremor (WHIGET) Rating Scale;	Observational study	No records found
Zetterberg, et al. 2015[39]	to To explore components of passive movement resistance in the wrist and finger muscles using NeuroFlexor method in PD.	YES	PD motor symptoms; Bradykinesia; Rigidity; Tremor; Stretch; Movement resistance	PD: 72.3<br>CG: 4	1–4	PD: 7<br>CG: NA	PD: 25<br>CG: 14	ON	A cross-sectional comparison was performed in twenty-five subjects with PD with clinically identified rigidity and 14 controls. Neural (NC), elastic (EC), and viscous (VC) components of the resistance to passive extension of the wrist were calculated using the NeuroFlexor.	No follow-up	UPDRS III; NeuroFlexor (Contributing to Passive Movement Resistance Contralateral Activation Maneuver)	Exploratory Study	No records found
Abbruzzese et al. 2014[69]	To review the available evidence on the effects of proprioceptive stimulation in improving UL mobility in patients with Movement disorders, and highlights the emerging innovative approaches targeted to maximizing the benefits of exercise by means of enhanced proprioception.		Somatosensory abnormalities; Proprioception	Not Mentioned	Not Mentioned		N: 20<br>PD: 12	Not mentioned	This perspective article focuses primarily on the rationale and the available evidence of the effects of rehabilitative strategies based on *proprioceptive training* in improving upper limb mobility of patients with MDs	No follow-up	Ectromyography (EMG); Biofeedback;	Clinical perspective	No records found
Sterlin et al. 2015	To investigate the effects of dopaminergic drugs on arm swing velocity, symmetry, and coordination in PD.	NO	PD symptoms; Tremor, Rigidity; Arm swing (velocity, symmetry, and coordination)	PD: 64.2<br>CG: 61.8	3–4	PD: 2.7<br>CG: NA	PD: 16<br>CG: 17	ON and OFF	Angular velocity of arm swing was recorded using wearable triaxial inertial sensors	No follow-up	UPDRS-III; APDM Emerald (Arm swing measurements)	Experimental study	No records found
Capato et al. 2019[8]	To present the effects of internal and external compensation strategies in a patient with both FOG and FOUL.	YES	PD symptoms; FOUL;	PD: 64	4	PD: 30	PD:1	ON	External compensations strategies with and without auditory cues; internal attentional compensation strategies.	NA	MDS-UPDRS-III; Spiral test; Funnel task.	Case report	No records found
Kueper et al. 2017[63]	To assess whether there is an association between performance on motor function tests and incident dementiaincliding PD.	YES	Upper limb motor function; Manual Dexterity; Tremor; Bradykinesia; Rigidity; Finger Tapping; PD motor symptoms	NA	NA	NA	N = 37	NA	Electronic database, gray literature and hand searching identified studies testing for associations between baseline motor function and incident dementia in older adults including PD	No follow-up	PPT; Finger Tap test	Systematic review and meta-analysis	No records found

(*Continued*)

 © 2023. Academia Brasileira de Neurologia. All rights reserved.

Supplementary Material Table 1 (*Continued*)

Author	Objective	UL impairments as an Inclusion Criteria	Upper limb impairments assessed	Age (Mean)	H&Y	Disease duration (years) mean	Sample	Medication state (ON/OFF)	Intervention	Follow-up	Outcomes to access Upper limb	Study design	PEDro (0/10)
Knippenberg et al. 2017[67]	To investigate which motion capture systems are used as training devices in neurological rehabilitation including PD; how they are applied; in which target population; what the content of the training; efficacy of training with mcs is.	NO	Not mentioned	NA	NA	NA	N: 66 (03 PD)	NA	Motion capture systems (mcs); gesture xtreme capture platform, standard laptop and a low-cost webcam. Studies used standardized exercises or games, with respect to therapeutic goals and focused on the impaired body part or functionality, but never involving the patient in the process	NA	Not mentioned	Systematic review	No records found
Moumdjian et al. 2017[47]	To describe and define music-based intervention modalities and content which are applied in experimental studies; to describe the effects of these interventions on motor and/or cognitive symptoms in the neurological population including PD.	NO	PD motor symptoms; Manual Dexterity; Strength;	NA	NA	NA	N:19 (03 PD)	NA	Measures used to investigate intervention effects were the raw pre- and post-values inclusive of their SDs, statistical significance of intervention, and group by time interactions.	NA	UPDRS III; Box and block test; NHPT; grip and pinch dynamometer; computerized movement analysis; computerized movement analysis;	Systematic Review	No records found
Proud et al. 2015[16]	To identify measurement tools used for UL evaluation PD; to summarize the content of each tool using the International Classification of Functioning, Disability and Health; to examine the reliability, validity, clinical utility, and responsiveness of the measurement tools specific to this clinical group.	YES	PD motor symptoms; Tremor; Bradykinesia; Manual Dexterity; Strength;	NA	NA	NA	N: 18	NA	The review was conducted in 2 parts: an initial search to identify upper limb measures described in the PD literature and a second search to retrieve studies investigating the measurement properties of these tools in this clinical group.	No follow up	MDS-UPDRS; UDPRS III; NHPT; Box and Block Test; CRT; PPT; Arm, Shoulder and Hand (DASH) questionnaire; CAPSIT-PD; Modified Bradykinesia Rating Scale; Action Research Arm Test; Fugl-Meyer scale; Grooved Pegboard Test; Minnesota Manual Dexterity Test; Motor Performance Series.	Systematic Review	No records found
Ryan d., et al. 2021[41]	To investigate the effect of action observation therapy (AOT) in the rehabilitation of neurologic(including PD) and musculoskeletal conditions.	NO	PD motor symptoms;	NA	NA	NA	In total 36 studies were included; 32 studies evaluated neurologic conditions: cerebral palsy ($n=6$), dementia ($n=1$), multiple sclerosis ($n=1$), Parkinson disease ($n=5$), or stroke ($n=19$; and 04 . musculoskeletal conditions.	NA	NA	NA	International Classification of Functioning, Disability, and Health (ICF); UPDRS	Systematic Review	Systematic review (N/A)
França, et al. 2018[42]	To review the current evidence for cerebellar modulation in movement disorders and its safety profile.	NO	Dyskinesia; Manual Dexetery	NA	NA	NA	34 studies were included; comprising 431participnats.	NA	Eligible studies were identified after a systematic literature review of the effects of cerebellar modulation in cerebellar ataxia, PD, ET dystonia and Progressive supranuclear palsy . Neuromodulation techniques included transcranial magnetic stimulation (TMS), transcranial direct current stimulation (tDCS) and deep brain stimulation (DBS). The changes in motor scores and the incidence of adverse events after the stimulation were reviewed.	NA	UPDRS; UPDRS IV	Systematic Review	No records found
Simpson et al. 2020[44]	To systematically establish the effects of tDCS on upper limb motor performance in PD patients.	YES	PD motor symptoms; Manual Dexterity; Reaction time; FOUL	58–74	2–3	5.8 - 12.3	N: 10	ON and OFF	Six databases were systematically searched for full-text articles published in English	NA	UPDRS III; PPT; Finger tapping, Motor-evoked potential, T active motor threshold, Reaction time, Hand Sup/Pro supination and pronation, UL-MT upper limb motor task	Systematic Review	No records found
Lora-Millan et al. 2021[45]	To review the devices developed to tremor management paradigms; to evaluates the different metrics used for the	YES	Tremor	NA	NA	NA	N: 36	NA	Literature search using three different databases. Several aspects of the selected documents were analyzed: (i) working principle	NA	Spiral Test; handwriting and drawing patterns using a digitizing tablet. UPDRS; FTM; TETRAS	Review	No records found

 © 2023. Academia Brasileira de Neurologia. All rights reserved.

Supplementary Material Table 1 (*Continued*)

Author	Objective	UL impairments as an Inclusion Criteria	Upper limb impairments assessed	Age (Mean)	H&Y	Disease duration (years) mean	Sample	Medication state (ON/OFF)	Intervention	Follow-up	Outcomes to access Upper limb	Study design	PEDro (0/10)
	validation of these devices and the lack of a standard validation procedure that allows the comparison among them.								and hypothesis that supported the tremor management approach for each device; (ii) the experimental setup for device validation; (iii) subject sample size, tremor pathology, and metrics used to quantify the tremor reduction; and (iv) the efficacy of the tremor suppression reported by each approach.				
Mittal et al. 2020[46]	To identify novel objective techniques through which botulinum toxin injections reduced ET or PD tremor effectively yet caused less weakness compare with the previously reported methodology.	YES	Tremor;; Hand strength; Patient perception of tremor	Not Mentioned	Not Mentioned	Not Mentioned	NTotal: 16<br>NPD: 6<br>ET:10	ON	An electronic search was performed on Medline using "Tremor," "Essential Tremor" and "Parkinson disease tremor" as keywords. Each of these, were crossed with "Botulinum Toxin OR Botulinum Neurotoxin"; publications were classified according to the criteria of the American Academy of Neurology.	No follow-up	UPDRS; FTM; Writing and spiral drawing; Patient Global Impression of Change; PDQLS	Review	No records found

Abbreviations: H&Y, Hoehn & Yahr Parkinson's scale; NHPT,-Nine-Hole Peg Test; PPT, Purdue Pegboard Test; MDS-UPDRS, Movement Disorder Society-sponsored revision of the Unified Parkinson's Disease Rating Scale; UL-UPDRS-III, Modified Unified PD Rating Scale motor scale including all items concerning upper limb motor function; UPDRS, Unified Parkinson's Disease Rating Scale; QUEST, Quality of Life for Essential Tremor Questionnaire; FTM, Fahn-Tolosa-Marin tremor rating scale; QoL, Quality of life; VAS, Visual analogue scale; PDQ-39, Parkinson Disease Questionnaire-39; DextQ-24, Patient-reported dexterity questionnaire; CRT, Coin rotation task; FR, Functional Reach; COTNAB, Chessington Occupational Therapy Neurologic Assessment Battery; ARAT, Action Research Arm Test; BBT, Box and Block Test; CRST, Clinical Rating Scale for Tremor; FMA, Fugl-Meyer Assessment; MMDT, Minnesota Manual Dexterity Test; MPS, Motor Performance Series; CAPSIT-PD, Core Assessment Program for Surgical Interventional Therapies in Parkinson's disease; CRST, Clinical Rating Scale for Tremor; COPM, Canadian Occupational Performance Measure; GAS, Goal Attainment Scale; JTHF, Jebsen Test of Hand Function; DASH, Disabilities of the Arm, Shoulder and Hand; TETRAS, Tremor Rating Assessment Scale; SPDDS, Self-assessment Parkinson's Disease Disability Scale; WHIGET, Washington Heights–Inwood Genetic Study of Essential Tremor; Funnel task, Edinburgh Handedness Inventory.

 © 2023. Academia Brasileira de Neurologia. All rights reserved.
